# Management of Coronal Dens Invaginatus Using Nonsurgical and Surgical Approaches Assisted by a 3D-Printed Model: A Case Report

**DOI:** 10.7759/cureus.95367

**Published:** 2025-10-25

**Authors:** Kiran Kumar, Ramya Kiran, Yu Yao Teoh, George Bogen

**Affiliations:** 1 Endodontics, School of Dentistry, The University of Queensland, Brisbane, AUS; 2 Dentistry, School of Dentistry, The University of Queensland, Brisbane, AUS

**Keywords:** 3d printing, dens in dente, dens invaginatus, mineral trioxide aggregate(mta), surgical endodontics

## Abstract

Coronal dens invaginatus (DI) is a developmental anatomical anomaly that forms during odontogenesis. A 12-year-old female presented to our clinic with a labial draining sinus tract associated with the maxillary right lateral incisor, which had persisted for several months. The patient reported no additional symptoms. Radiographic evaluation revealed a mid-radicular osseous defect involving the affected tooth. Cone beam CT (CBCT) confirmed the presence of a developmental anomaly, classified as Type 3a DI according to Oehlers’ classification. Pulp sensibility testing of the lateral incisor was inconclusive, whereas all adjacent teeth responded normally. The tooth initially underwent nonsurgical endodontic treatment involving both the primary root canal and the invaginated canal. Treatment commenced after identifying a hypoplastic dentinal bridge separating the DI from the main canal. Management included placement of a mineral trioxide aggregate plug at the apex of the rudimentary root, which exhibited aberrant morphology. Although the draining sinus initially resolved following nonsurgical endodontic therapy, it recurred at the 12-month follow-up. Surgical treatment was then undertaken after generating a 3D-printed model to better understand the complexity and orientation of the anatomical anomaly, guided by CBCT imaging. Clinical review after surgical endodontic treatment at 12 months revealed resolution of the draining sinus. Radiographic evaluation showed a reduction in radiolucency, and follow-up at three years and 10 months demonstrated complete osseous repair of the latero-radicular lesion. Successful management of this case required both nonsurgical and surgical endodontic interventions. The incorporation of 3D printing facilitated surgical planning and contributed to improved treatment outcomes in teeth exhibiting complex anomalies such as DI.

## Introduction

Dens invaginatus (DI) is a developmental dental anomaly most commonly reported in maxillary lateral incisors, with a prevalence ranging from 0.3% to 10% and a female predilection of approximately 3:1 [1,2]. It is classified based on the location and origin of the invagination into coronal and radicular variants. While the exact etiology remains unclear, potential contributing factors include growth pressure from the dental arch, focal growth failures of the internal enamel epithelium, distortion of the enamel organ during tooth development, fusion during gemination, infection, and trauma [3].

Oehlers classified coronal DI into three types [4,5]. Type 1 is confined to the crown and does not extend beyond the cemento-enamel junction (CEJ). Type 2 extends beyond the CEJ but remains within the root, with no periodontal communication. Type 3 extends through the root and is further subdivided based on the location of the opening of the invagination along the root. In type 3a, the invagination communicates laterally with the periodontal ligament, while in type 3b, it communicates apically. The coronal variant results from the infolding of the enamel organ in the crown, which appears as a pit or groove and leads to atypical tooth morphology.

Radicular DI is classified into two types [6]. Type 1 involves axial infolding of a root wall, representing an incomplete attempt at root bifurcation. The root tapers to a blunt apex, with a deep axial infolding of the lingual wall producing a horseshoe-shaped cross-section. Type 2 arises from a foramen on the root surface, forming a saccular, dilated invagination within an expanded root, lined internally by enamel rather than cementum.

Teeth with DI may exhibit unusual morphologies such as peg-shaped, conical, or barrel-shaped forms, or appear normal with localized hypoplastic areas [1]. Histologically, invaginated tissues may differ from normal enamel and dentin, showing irregular, hypomineralized structures with altered composition [7]. The porous nature of the invagination can provide a route for bacterial ingress, potentially leading to pulp necrosis. Diagnosis of pulp vitality can be challenging, as teeth may respond to sensibility tests despite the presence of periapical radiolucency [8]. In some cases, vital pulp tissue persists even in the presence of periapical pathology, likely due to direct communication between the pulp space and periradicular tissues, allowing microbial entry without immediate compromise of pulp vitality [9].

Management strategies for DI include regular radiographic monitoring, fissure sealants, prophylactic intervention, conventional root canal therapy, and surgical treatment [1,3,10]. Historically, extraction was the primary treatment for pathologic or complex DI prior to the 1970s [1]. Challenges in treatment include identifying primary and secondary canal communications, removing internal anatomical structures, and effectively instrumenting, debriding, and obturating large or atypical canal spaces [3]. Surgical intervention is typically reserved for cases refractory to nonsurgical treatment, particularly when apical sealing is not possible [10]. Cone beam CT (CBCT) provides 3D visualization, aiding in precise anatomical assessment and treatment planning [11]. Advances such as CBCT, dental operating microscopes, ultrasonication, and calcium silicate cements have significantly improved treatment outcomes [11-13].

This case report describes the successful management of an Oehlers type 3a coronal DI in a maxillary right lateral incisor of a young patient, using a 3D-printed model to assist with surgical planning [4-6]. The tooth initially presented with a latero-radicular lesion managed nonsurgically, but recurrent symptoms necessitated surgical intervention. The 3D-printed model allowed for a detailed understanding of the complex anatomy prior to surgery.

## Case presentation

Nonsurgical treatment

**Figure 1:**
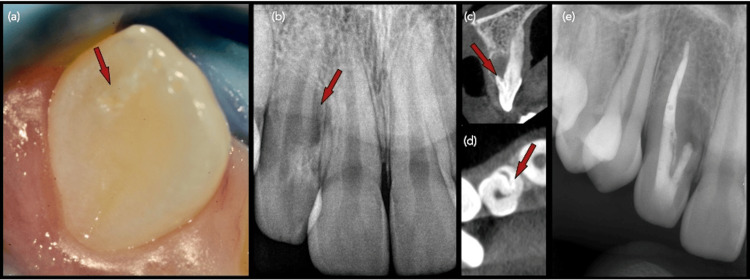
(a) Pretreatment clinical image of the right maxillary lateral incisor (#12) showing a hypoplastic enamel area on the mesio-incisal half of the palatal surface (arrow) (×6.3). (b) Pretreatment periapical radiograph revealing a mid-radicular radiolucency (arrow) and atypical anatomy at the cementoenamel junction. (c) Sagittal CBCT image of #12 demonstrating a rudimentary root on the labial aspect (arrow) and an associated osseous defect extending to the mid-root and buccal plate. (d) Axial CBCT section showing a mesial fissure of the main canal (arrow) leading to the accessory root. (e) Immediate post-nonsurgical endodontic treatment radiograph illustrating the distal main canal obturated with gutta-percha and AH Plus sealer, while the mesial accessory canal was sealed with an MTA apical plug. CBCT, cone beam CT; MPA, mineral trioxide aggregate

A 12-year-old female patient was referred by her general dentist for management of a draining sinus associated with the right maxillary lateral incisor (tooth #12). Extraoral examination was unremarkable. Intraorally, the patient presented with mixed dentition and mild crowding. Clinical examination revealed a sinus tract opening in the labial vestibule near the mid-root of tooth #12. Additionally, a 2 × 2 mm enamel hypoplastic defect was observed on the mesiopalatal surface of #12 (Figure 1a), measured using a William’s periodontal probe.

The tooth was non-carious and had no known history of trauma. All other maxillary anterior teeth displayed normal morphology and probing depths and exhibited normal mobility, including the contralateral lateral incisor (tooth #22). Tooth #12 was nonresponsive to percussion testing, though mild tenderness was noted on palpation of the labial gingiva adjacent to the tooth. Pulp sensibility testing yielded inconclusive results, whereas all adjacent maxillary anterior teeth responded positively to cold testing (Endo Frost, Coltene Whaledent Roeko, Langenau, Germany).

Radiographic examination revealed a large radiolucency on the mesial aspect of the mid-root region of tooth #12, associated with atypical radicular morphology. A small field-of-view CBCT scan (Morita 3D Accuitomo 170, J. Morita, Saitama, Japan) was performed with the following parameters: 100 kVp tube voltage, 10 mA tube current, voxel size of 0.20 × 0.20 × 0.20 mm, and an exposure time of 9.4 seconds. The scan confirmed aberrant radicular morphology consistent with DI, characterized by a primary root and an accessory rudimentary root visible in sagittal sections (Figure 1b). Sagittal CBCT imaging also revealed the accessory root positioned on the labial aspect, with an associated osseous defect extending to the mid-root region and involving the buccal cortical plate (Figure 1c). Axial CBCT sections demonstrated a fissure originating from the mesial aspect of the main canal, extending toward and communicating with the accessory root (Figure 1d). The accessory root displayed incomplete development with radiographic features indicative of an open apex. Further evaluation confirmed a mesial canal fissure leading directly to the vestigial root. Based on the presence of a draining sinus tract and pulp sensibility test results, tooth #12 was diagnosed with pulp necrosis and a chronic periapical abscess. After discussing risks, benefits, and expected outcomes, parental consent was obtained for conventional orthograde endodontic treatment.

Local anesthesia was administered following topical application of 2% lignocaine, using 2% lignocaine with 1:80,000 adrenaline (Lignospan, Septodont, Saint-Maur-des-Fossés, France). A dental dam (Hu-Friedy, Chicago, IL, USA) was placed to ensure isolation, and endodontic access was initiated through the palatal hypoplastic area of tooth #12 under magnification with a dental operating microscope (DOM, Carl Zeiss, Oberkochen, Germany). A deep groove was observed in the accessory root canal communicating with the main canal. The dentinal wall separating the DI and main canal appeared porous under magnification, and the decision was made to treat both canals simultaneously. Both canals were shaped using rotary files to size #25 with variable taper (Protaper^®^ Next X2, Dentsply Sirona, Ballaigues, Switzerland) and irrigated with 4% sodium hypochlorite, followed by a final rinse with 15% EDTA (Dentalife Pty Ltd, Ringwood, Australia). The main canal measured 23 mm, and the accessory root canal measured 12 mm.

Due to apical hemorrhage, the DI was obturated using mineral trioxide aggregate (MTA) to create an apical plug (ProRoot^®^ MTA, Maillefer, Johnson City, TN, USA). The main canal was obturated with warm vertically compacted gutta-percha and epoxy-resin sealer (AH Plus^®^, Dentsply Maillefer, Johnson City, TN, USA) (Figure 1e). The access cavity was restored with composite resin (3M Filtek Supreme XTE Universal, St. Paul, MN, USA).

**Figure 2:**
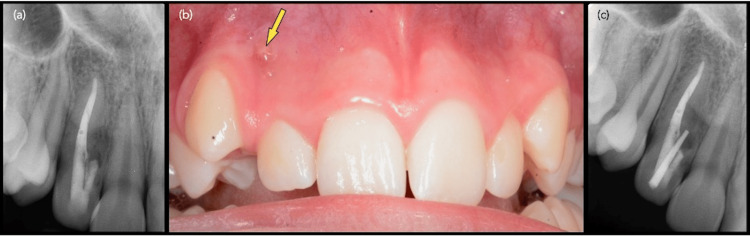
(a) Twelve-month periapical radiograph showing a persistent mid-radicular radiolucency. (b) Intraoral photograph at 12-month recall after nonsurgical root canal treatment, revealing an active draining sinus tract on the mid-buccal plate between teeth #12 and #13 (arrow) (×6.3). (c) Radiograph with gutta-percha point tracing the sinus tract toward the mesial radiolucent area.

At the four-week recall, the draining sinus tract had resolved, and the patient was asymptomatic. However, at the 12-month follow-up, the sinus tract had recurred and was associated with a persistent radiolucency (Figure 2a, 2b). A modified ISO size 20 gutta-percha point (Dentsply Maillefer) was inserted through the sinus tract on the buccal plate and traced to the radiolucent area (Figure 2c). The parents were informed of the complex root anatomy of tooth #12 and the need for further treatment, and informed consent was obtained for the planned surgical endodontic procedure.

Surgical treatment

**Figure 3:**
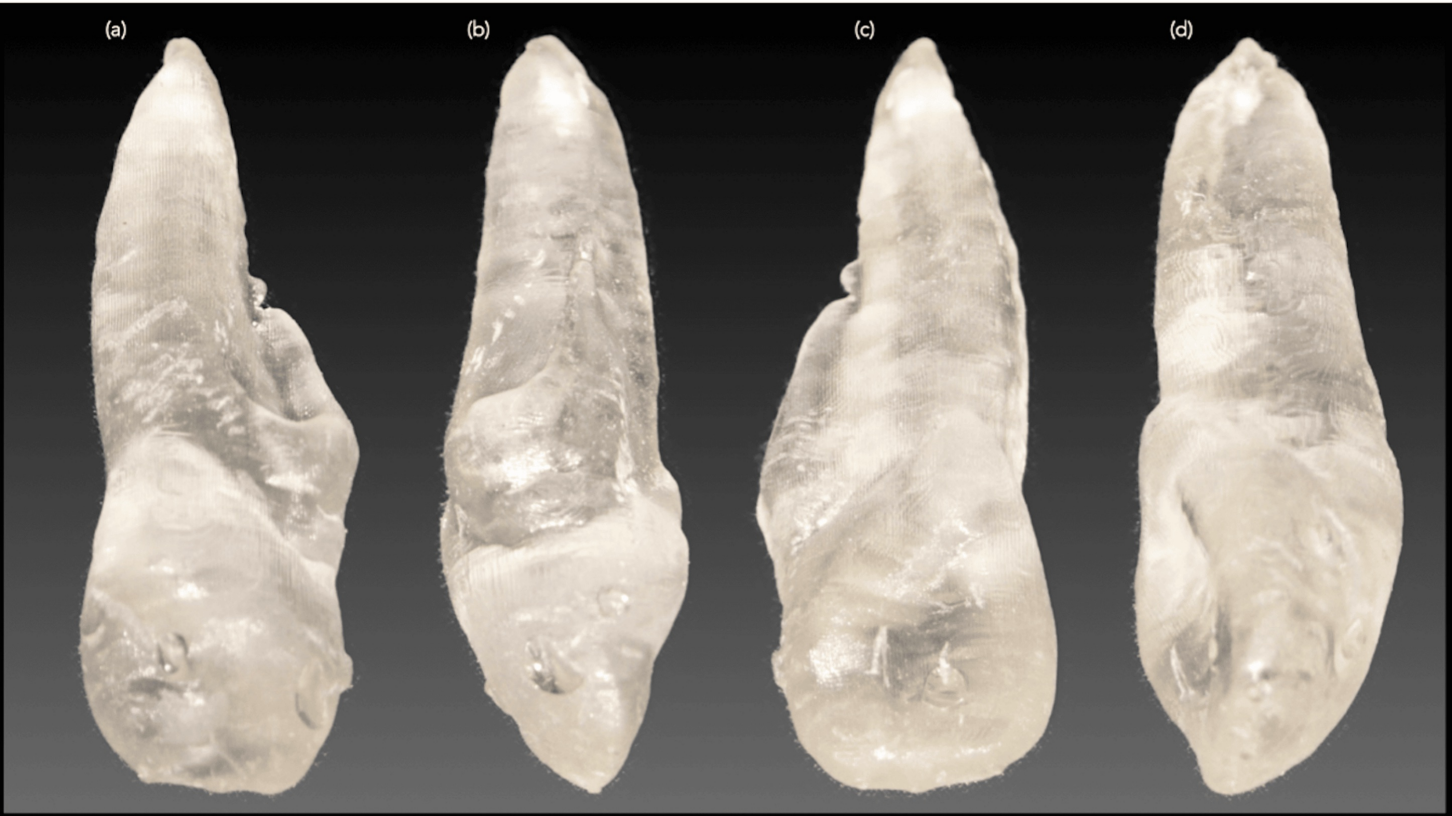
Photographs of the 3D printed resin model showing (a) labial, (b) mesial, (c) palatal, and (d) distal views.

Prior to surgical treatment, CBCT data were used to generate a 3D rendering of the tooth, which was then employed to produce an isometric 3D resin model (Figure 3) using free, open-source software (www.slicer.org) and printed with Vertex Implacryl liquid/powder (Vertex Dental B.V., Soesterberg, The Netherlands). The 3D printed model was carefully examined to guide the surgical strategy, including planning the osteotomy size, orientation of the radicular anomaly, and the extent of root resection, using a William’s periodontal probe.

**Figure 4:**
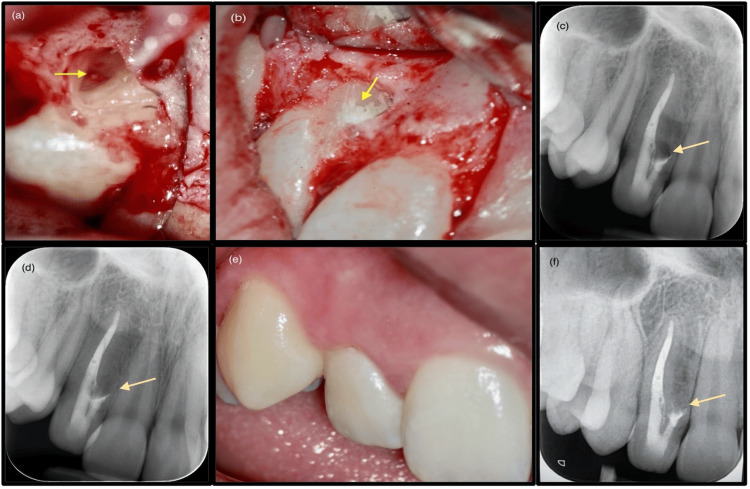
(a) Clinical photograph of tooth #12 after flap reflection, showing incomplete radicular formation near the cementoenamel junction and the mid-radicular defect with loss of the buccal plate (arrow) (×10). (b) Clinical photograph after resection and placement of MTA retrograde filling into the DI defect (arrow) (×10). (c) Immediate postoperative radiograph showing MTA retrofill/repair placement (arrow). (d) Twelve-month postoperative radiograph demonstrating reduction of the radiolucency (arrow). (e) Intraoral photograph at 12-month recall showing soft tissue healing and absence of a sinus tract. (f) Radiograph at three years and 10 months demonstrating complete osseous repair and mineralization of the defect (arrow). DI, dens invaginatus; MTA, mineral trioxide aggregate

After topical anesthesia with 2% lignocaine, local anesthesia was achieved using two cartridges of 2% lignocaine with 1:80,000 adrenaline (Lignospan, Septodont). A full-thickness mucoperiosteal flap was raised from the buccal plate, with releasing incisions placed distal to the canine and mesial to the maxillary right central incisor (Figure 4a). Inspection of the osseous defect revealed deficient radicular formation near the cementoenamel junction and complete loss of the buccal plate. Fibrous connective tissue covered the mid-radicular defect, extending over both roots proximal to tooth #12. Examination of the accessory root revealed root resorption, incomplete development, and an open apex.

The rudimentary root containing the DI was sequentially resected using a #703 carbide bur mounted on a high-speed surgical handpiece. A 2 mm apical root resection was performed to address the resorption. Retrograde cavity preparation was carried out using a diamond-coated ultrasonic tip (E32D, NSK Ltd, Tokyo, Japan), after which MTA was mixed according to the manufacturer’s instructions and placed as the retrograde filling material to ensure an effective seal and prevent potential microleakage (Figure 4b, 4c). A biopsy of the fibrous tissue from the osteotomy site was submitted for histological examination. The flap was repositioned and secured with 5/0 monofilament interrupted sutures (Dyloc, Dynek Pty Ltd, Port Adelaide, Australia). Healing was uneventful, with complete resolution of the sinus tract noted at the one-week postoperative suture removal.

Histopathological analysis revealed calcified deposits within benign fibro-myxoid stroma containing focal epithelial odontogenic rests. A definitive diagnosis could not be established due to nonspecific findings (Table 1). Radiographic review at 12 months (Figure 4d, 4e) demonstrated a reduction of the mid-radicular osseous defect with no recurrence of the draining sinus. At three years and 10 months, radiographs (Figure 4f) confirmed complete resolution. The patient remained asymptomatic, with the tooth firm and exhibiting normal mobility, probing depths, and function.

**Table 1:** Histopathology report

Parameter	Description
Patient ID	12-year-old female
Specimen	Periapical area of tooth 12
Clinical diagnosis	Chronic periapical abscess
Macroscopic findings	One piece, measuring 4 × 4 × 2 mm; processed in toto (1A)
Microscopic findings	The specimen consists of fibromyxoid stromal tissue containing abundant calcified debris. Focal epithelial odontogenic rests are present within the stroma, and one edge of the fragment shows a cuboidal epithelial lining suggestive of sampling from a dental follicle.
Histopathological diagnosis	Findings are nonspecific and difficult to interpret.

## Discussion

This case underscores the importance of long-term follow-up in endodontically treated teeth affected by DI, even when early clinical and radiographic findings suggest symptomatic resolution. The combination of absent overt symptoms, a persistent draining sinus tract, inconsistent responses to pulp sensibility testing, and a radiolucent area on the lateral root surface, accompanied by atypical radiographic features, posed significant diagnostic challenges. Clinically, the crown appeared largely normal except for a hypoplastic area on the palatal surface, visible only under magnification, which added to the complexity of diagnosis. Microbial ingress through this hypoplastic region likely contributed to pulpal necrosis and formation of the sinus tract [14]. Residual vital pulp tissue within the invaginated structure may result in false-positive sensibility test responses [8]. CBCT imaging provided critical anatomical information, revealing that the extensive invagination most likely originated coronal to the root. Nevertheless, accurate detection and classification of such anatomical anomalies can be challenging without detailed 3D imaging.

A key limitation in this case was the inability of conventional 2D radiographs to definitively assess treatment outcomes, particularly the complete resolution of periapical pathology. Traditional imaging lacks the spatial resolution and depth required to visualize subtle anatomical variations or healing changes. In contrast, CBCT offers precise 3D visualization, enhancing diagnosis, treatment planning, and follow-up, making it indispensable in complex cases. The unusual clinical presentation, including absent symptoms, a draining sinus tract, inconsistent sensibility responses, and a hypoplastic palatal defect, further complicated diagnosis. The presence of residual vital tissue within the invagination poses a risk of false-positive responses with conventional pulp testing, highlighting the need for more accurate vitality assessment methods, such as laser Doppler flowmetry or pulse oximetry, which evaluate vascular perfusion rather than neural response.

3D printing can serve as a powerful diagnostic tool when the printed model accurately replicates the imaged structure [15]. In endodontics, 3D printing has been applied for guided endodontic access and endodontic microsurgery [15,16]. In this case, the 3D printed model of the tooth helped the clinician understand the anatomical complexities prior to surgical intervention. Careful examination of the model revealed the presence and orientation of a rudimentary accessory root and the radicular groove, allowing for improved preoperative planning and reducing total surgical time by enhancing comprehension of the internal and external tooth anatomy [15].

Cases of DI have been successfully managed using both nonsurgical and surgical endodontic approaches [9,10]. Treatment may target the DI area in isolation or include the main canal simultaneously [3]. Successful management requires the complete removal of irritants from the root canal system, which is particularly challenging in cases with complex anatomy. Adequate cleaning also necessitates sufficient irrigation to debride the canal, a process made more difficult in teeth with open or resorbed apices. Orthograde placement of MTA as an apical barrier in teeth with open or immature apices has been reported to yield favorable outcomes [17]. However, in this case, the initial MTA apical plug failed to promote remineralization and repair of the periodontium. MTA requires mixing, which may lead to material wastage and discoloration due to bismuth oxide [18]. Premixed bioceramic putty, offering similar properties to MTA, is now available and overcomes disadvantages such as discoloration, delayed setting, and the need for mixing [19].

Small, isolated voids were noted in the obturation. While these may not compromise treatment success when coronal sealing and disinfection are adequate, multiple or extensive voids can increase the risk of failure. Therefore, minimizing voids is essential to ensure long-term success and maintain a biological seal.

The patient’s young age was an additional critical consideration, influencing the decision to avoid primary surgical intervention. This choice considered potential postoperative aesthetic complications, including loss of interdental papilla, increased tooth morbidity, and risk of horizontal alveolar bone loss. MTA was chosen as the obturation material and applied as an apical barrier to seal the incompletely closed apex resulting from the DI morphology. Despite this, orthograde MTA obturation did not achieve complete tissue repair, necessitating surgical treatment. The 12-month post-surgical review demonstrated resolution of the draining sinus, with radiographs at 12 months showing reduced radiolucency and at three years and 10 months confirming complete healing of the latero-radicular lesion. CBCT was not performed at recall due to circumstances beyond the operator’s control.

The clinical relevance of this case lies in highlighting both the diagnostic complexity and therapeutic implications of DI. Early detection and appropriate imaging are crucial to prevent the progression of undiagnosed pulpal or periapical pathology, which could otherwise lead to extensive destruction or tooth loss. Looking ahead, AI has promising potential in endodontics. AI-driven algorithms may assist in automated detection and classification of anatomical anomalies on CBCT scans, improve diagnostic accuracy, and support clinical decision-making. Machine learning models trained on large datasets could one day predict treatment outcomes or identify early pathological changes not readily visible to the human eye.

The etiological basis of the primary infection in this case is likely multifactorial. Potential pathways include bacterial ingress via the hypoplastic palato-incisal area of the crown or pathosis originating from a periodontal source along the radicular groove. However, initial probing under local anesthesia did not reveal any clinical periodontal defects. Possible causes of nonhealing after initial orthograde treatment include the complex anatomical characteristics of the DI, infection of periodontal origin, or residual microorganisms protected by biofilm formation [20].

## Conclusions

DI represents a relatively common coronal malformation arising during tooth development and can pose significant diagnostic and therapeutic challenges. In this case, particularly given the patient’s young age, both nonsurgical and surgical interventions were necessary to achieve successful management and resolution of the pathosis. The combined use of CBCT imaging and 3D printing of the anatomical anomaly facilitated treatment planning, enhanced surgical predictability, and ultimately contributed to favorable healing outcomes in a tooth with complex morphology.

## References

[1] Hülsmann M (1997). Dens invaginatus: aetiology, classification, prevalence, diagnosis, and treatment considerations. Int Endod J.

[2] Alani A, Bishop K (2008). Dens invaginatus. Part 1: classification, prevalence and aetiology. Int Endod J.

[3] Bishop K, Alani A (2008). Dens invaginatus. Part 2: clinical, radiographic features and management options. Int Endod J.

[4] Oehlers FA (1957). Dens invaginatus (dilated composite odontome): II. Associated posterior crown forms and pathogenesis. Oral Surg Oral Med Oral Pathol.

[5] Oehlers FA (1957). Dens invaginatus (dilated composite odontome): I. Variations of the invagination process and associated anterior crown forms. Oral Surg Oral Med Oral Pathol.

[6] Oehlers FA (1958). The radicular variety of dens invaginatus. Oral Surg Oral Med Oral Pathol.

[7] Beynon AD (1982). Developing dens invaginatus (dens in dente). A quantitative microradiographic study and a reconsideration of the histogenesis of this condition. Br Dent J.

[8] Gonçalves A, Gonçalves M, Oliveira DP, Gonçalves N (2002). Dens invaginatus type III: report of a case and 10-year radiographic follow-up. Int Endod J.

[9] Tsurumachi T (2004). Endodontic treatment of an invaginated maxillary lateral incisor with a periradicular lesion and a healthy pulp. Int Endod J.

[10] Ozbas H, Subay RK, Ordulu M (2010). Surgical retreatment of an invaginated maxillary central incisor following overfilled endodontic treatment: a case report. Eur J Dent.

[11] Decurcio D, Silva J, Decurcio RA, Silva RG, Pécora JD (2011). Influence of cone beam computed tomography on dens invaginatus treatment planning. Dent Press Endod.

[12] Khalighinejad N, Aminoshariae A, Kulild JC, Williams KA, Wang J, Mickel A (2017). The effect of the dental operating microscope on the outcome of nonsurgical root canal treatment: a retrospective case-control study. J Endod.

[13] Plotino G, Pameijer CH, Grande NM, Somma F (2007). Ultrasonics in endodontics: a review of the literature. J Endod.

[14] Siqueira JF Jr, Rôças IN (2011). Microbiology and treatment of endodontic infections. Cohen's Pathways of the Pulp (Tenth Edition).

[15] Liu Y, Liao W, Jin G, Yang Q, Peng W (2014). Additive manufacturing and digital design assisted precise apicoectomy: a case study. Rapid Prototyp J.

[16] Connert T, Zehnder MS, Amato M, Weiger R, Kühl S, Krastl G (2018). Microguided endodontics: a method to achieve minimally invasive access cavity preparation and root canal location in mandibular incisors using a novel computer-guided technique. Int Endod J.

[17] Mente J, Leo M, Panagidis D, Ohle M, Schneider S, Lorenzo Bermejo J, Pfefferle T (2013). Treatment outcome of mineral trioxide aggregate in open apex teeth. J Endod.

[18] Torabinejad M, Parirokh M, Dummer PM (2018). Mineral trioxide aggregate and other bioactive endodontic cements: an updated overview - part II: other clinical applications and complications. Int Endod J.

[19] Debelian G, Trope M (2016). The use of premixed bioceramic materials in endodontics. G Ital Endod.

[20] Ricucci D, Siqueira JF Jr (2010). Biofilms and apical periodontitis: study of prevalence and association with clinical and histopathologic findings. J Endod.

